# Exploiting On-Chip Voltage Regulators for Leakage Reduction in Hardware Masking

**DOI:** 10.3390/s22187028

**Published:** 2022-09-16

**Authors:** Soner Seçkiner, Selçuk Köse

**Affiliations:** Department of Electrical and Computer Engineering, University of Rochester, Rochester, NY 14627, USA

**Keywords:** hardware masking, side-channel attack, voltage regulator, power delivery network, lightweight countermeasure

## Abstract

A design space exploration of the countermeasures for hardware masking is proposed in this paper. The assumption of independence among shares used in hardware masking can be violated in practical designs. Recently, the security impact of noise coupling among multiple masking shares has been demonstrated both in practical FPGA implementations and with extensive transistor level simulations. Due to the highly sophisticated interactions in modern VLSI circuits, the interactions among multiple masking shares are quite challenging to model and thus information leakage from one share to another through noise coupling is difficult to mitigate. In this paper, the implications of utilizing on-chip voltage regulators to minimize the coupling among multiple masking shares through a shared power delivery network (PDN) are investigated. Specifically, different voltage regulator configurations where the power is delivered to different shares through various configurations are investigated. The placement of a voltage regulator relative to the masking shares is demonstrated to a have a significant impact on the coupling between masking shares. A PDN consisting of two shares is simulated with an ideal voltage regulator, strong DLDO, normal DLDO, weak DLDO, two DLDOs, and two DLDOs with 180${}^{\circ }$ phase shift. An 18 × 18 grid PDN with a normal DLDO is simulated to demonstrate the effect of PDN impedance on security. The security analysis is performed using correlation and *t*-test analyses where a low correlation between shares can be inferred as security improvement and a *t*-test value below 4.5 means that the shares have negligible coupling, and thus the proposed method is secure. In certain cases, the proposed techniques achieve up to an 80% reduction in the correlation between masking shares. The PDN with two DLDOs and two-phase DLDO with 180${}^{\circ }$ phase shift achieve satisfactory security levels since *t*-test values remain under 4.5 with 100,000 traces of simulations. The security of the PDN improves if DLDO is placed closer to any one of the masking shares.

## 1. Introduction

Modern computing devices consist of various circuit components to perform different tasks. The security and privacy of data processed and stored in these devices have become important with the proliferation of modern computing devices in our daily lives. Cryptographic modules that perform encryption/decryption operations are therefore utilized to improve the security and privacy of data. To perform the encryption/decryption in a lightweight, fast, and power efficient manner, various algorithms with unique implementations have been proposed. However, side-channel attacks still threaten the security of all of these cryptographic devices. Passive and non-invasive side-channel attacks use certain intermediate values of an encryption algorithm to obtain physical leakage signatures, correlate this leakage with certain predetermined models, and eventually determine the private keys or passwords stored in these devices. To protect private data, different types of countermeasures have been developed [1,2]. The working principle of countermeasures against side-channel attacks can be broadly categorized into two: (i) shuffle and (ii) hide the private data. Masking based countermeasures are developed to shuffle the private data within the device by splitting an n-bit secret into N shares, similar to multi party computation.

The hiding countermeasures are difficult to implement since strict requirements such as aligned signal propagation and balanced routing are difficult to achieve at advanced technology nodes due to the increase in parasitic effects [3]. An efficient preprocessing and machine learning technique can reveal the information from an encryption device designed with weak countermeasures. Among other countermeasures, hardware masking typically provides a sufficient level of security against various attack types due to the robust design of masking supported by theory [4].

Masking divides the sensitive information into a d+1 share for a *d*th order Boolean masking where the sensitive information is the Boolean addition of each share. The operations in each share are unmasked and typical *d*th order masking can be defeated by (d+1)th order attack. The main assumption of a successful masking is that each share of a masking operation is independent. This assumption is so critical that the shares, otherwise, leak information due to the dependent statistical moments of each share, leading to a *d*th order attack to be successful on an encryption device with *d*th order masking. While hardware masking provides security by processing the sensitive data into multiple shares, the violation of the independence can lead to severe security vulnerabilities [5]. The masking can be implemented in software or hardware. Software implementation of hardware masking is naturally sequential and may be highly costly because of the high code size and long execution times [6]. On the other hand, hardware masking is highly flexible due to the parallel nature of hardware implementation and is highly suitable for high performance applications.

The practical implementations of hardware masking have certain challenges due to the parasitic impedances, and variations in the transistors and interconnections due to aging, temperature, or fabrication process, which make satisfying the independent masking share assumption quite difficult. The primary reasons for the gap between the theory and practice of the hardware masking due to the aforementioned design challenges are as follows: The Hamming distance leakage between hardware masking shares cannot be completely eliminated due to the shared architectural components between shares; the leakage between shares is dependent because of the nature of the chip manufacturing techniques; and the glitches propagate through the logic gates and between hardware masking shares. The interdependence of different shares of masking and potential countermeasures are studied in the literature [4,5,7,8,9,10,11,12,13].

There are a small number of papers that investigate the security vulnerability of hardware masking due to the violation of independence assumption. An ASIC (Application Specific Integrated Circuit) design framework is proposed in [14] to decrease the leakage between hardware masking shares. The framework implements a novel place and route strategy to reduce the leakage between the hardware masking shares. However, the power delivery effects are not studied in this work, and the leakage can be eliminated until 4 million traces, but the leakage can occur within 2k traces for the situations where the circuit has vulnerabilities. A 3D CMOS chip stacking technique is used to reduce the leakage in the power delivery network in [15]. This technology is implemented to reduce the leakage of the ASIC design methodology for the power delivery network; however, the problem of hardware masking is not studied in this work. The leakage within the power delivery network is reduced up to 18k traces. A road-map is provided to design a secure power delivery network for hardware masking in [16]. The design framework proposes certain design guidelines for secure hardware masking; however, the security of the proposed design guidelines has not been evaluated using actual masking shares with simulations, as we performed in this work.

The existing literature provides a limited number of solutions to the problem of hardware masking because the existing circuit design, placement, and routing tools for power delivery network do not typically consider security hardware masking as a design target and therefore have limited capacity to evaluate the security of the designs in the preliminary stage. Additionally, the evaluation of the leakage in early stages of the design still requires a large number of measurements which take considerable time using existing EDA tools. The practical implementation of any design can potentially have security vulnerabilities which are typically only evaluated after manufacturing. A cost and time effective way to minimize this leakage among masking shares is to design the power delivery network (PDN) and on-chip voltage regulator in a leakage-cognizant way. Accordingly, on-chip voltage regulators are utilized in this paper as a countermeasure to mitigate the leakage between hardware masking shares. A design space exploration of the implications of different voltage regulator topologies and placement techniques for the voltage regulators and masking shares is performed to demonstrate the effectiveness towards closing the gap between theory and practice for hardware masking implementations.

Motivation: Hardware masking aims to separate the sensitive information into multiple shares to improve the resistance against side-channel attacks [5]. The main strength of the hardware masking depends on the assumption of independence of each share. Theoretically, the leakage from a single share cannot be used to obtain the information in other shares. However, there are many cases for the practical implementations which endanger the independence assumption of the multiple shares, leading to information leakage between masking shares. The information leakage occurs due to multiple reasons including glitch in the gates, Hamming distance leakage [6], and non-independent leakage [13].

The non-independent leakage can be caused due to a shared PDN or certain logic circuitry [7] because voltage drop propagates through masking shares via a shared PDN. The logic cells are placed on a shared substrate, and the distributed logic cells are connected through a PDN. PDN is composed of an interconnection network, voltage regulators, and decoupling capacitors to distribute a robust supply voltage to various circuit components. The current demand from logic devices is supplied via a PDN. Parasitic resistance, capacitance, and inductance of the PDN can cause ground bounce and voltage drop (i.e., power/ground noise) [17]. Alternatively, logic core contains the functionality of the circuit. The logic core generally contains short wires and small parasitic resistances. However, there are coupling capacitors within the substrate due to the nature of a semiconductor, leading to crosstalk between individual logic blocks. As a result, these complex interactions jeopardize the independence assumption of the masking shares.

Our Contribution: Although there are many countermeasures against side-channel attacks, there are a limited number of these countermeasures that specifically focus on the vulnerability of hardware masking. The voltage fluctuations in PDN have been extensively investigated; however, security implications of noise for hardware masking have typically not been considered [18,19]. In addition, there are many papers [20,21,22] that use voltage regulators as a hiding countermeasure where the voltage regulators hide the power signatures from any suspicious adversary. However, our work focuses on improving the security aspects of hardware masking which shuffle the sensitive information by dividing them into masking shares. Moreover, our work uses the DLDO to improve the security where our previous works [21,22] use buck, LDO, and switch capacitor voltage regulators to improve the security with hiding the leakage signatures. To the best of our knowledge, there are limited works [14,15,16] that focus on the security vulnerability of hardware masking on ASIC design flow without considering on-chip voltage regulators. Therefore, we propose a lightweight integration of a countermeasure to improve the security of hardware masking utilizing voltage regulators. The proposed method can be applied to any hardware masking implementation within any kind of encryption algorithm.

First, for the first time, to the best of our knowledge, DLDO is used to improve the security vulnerability of hardware masking where DLDO inserts voltage fluctuations to improve the security of hardware masking. Second, we prove the methodology mentioned in [16] where the security improves with the distance of hardware masking shares increases. Third, a design space exploration of the implications of different voltage regulator topologies and placement techniques for the voltage regulators and hardware masking shares is performed and demonstrated the effectiveness of these techniques.

Paper Organization: The outline of the paper is provided as follows. A literature review/related works of on-chip voltage regulators and the specific voltage regulator that is used in this paper, and countermeasures against side-channel attacks are provided in Section 2. Theoretical modeling is explained in Section 3. The methodology followed throughout the paper is presented in Section 4. The verification and validation of the inputs are discussed in Section 5. The results are offered in Section 6. Finally, conclusions and future recommendations are drawn in Section 7.

## 2. Literature Review/Related Works

A literature review/related works is provided for digital low dropout (DLDO) voltage regulators in Section 2.1 and existing countermeasures against side-channel attacks in Section 2.2. Hardware masking is explained in detail in Section 2.3.

### 2.1. DLDO Voltage Regulators

**Figure 1:**
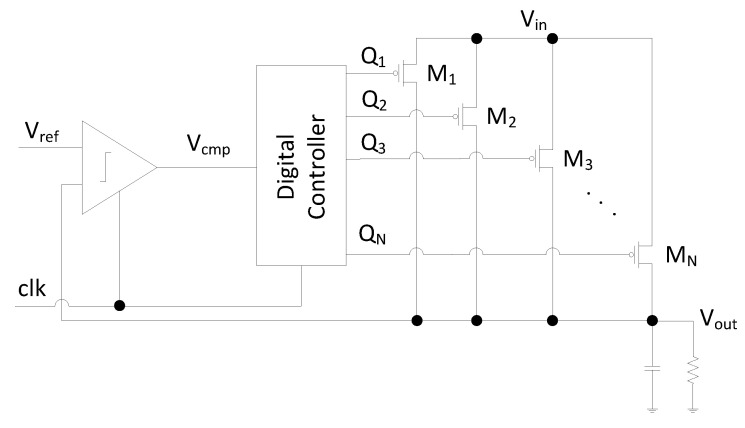
Schematic of a DLDO voltage regulator.

**Figure 2:**
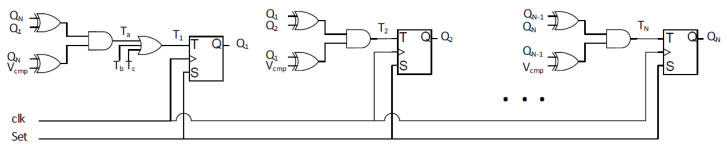
Schematic of the shift register utilized in the DLDO as used in [26,27].

**Figure 3:**
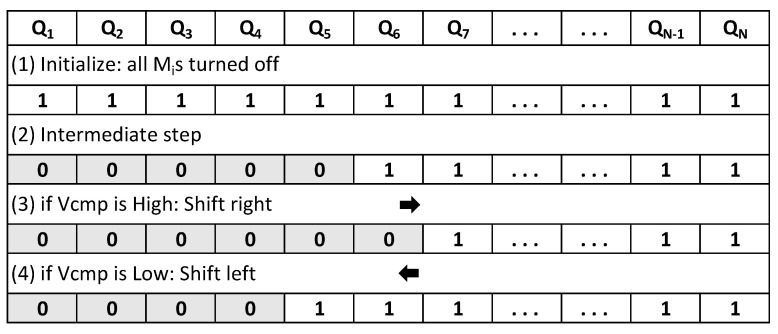
Operation principle of the shift register utilized in the DLDO.

Different types of voltage regulators can be utilized for fully on-chip implementations: low dropout (LDO), switched capacitor (SC), and buck voltage regulators [23,24]. Although utilizing any of these on-chip voltage regulators is expected to reduce the coupling among masking shares, a digital low dropout (DLDO) voltage regulator is utilized in this paper due to the ease of implementation, small area requirement, fast response time, and easy programmability. A schematic of a DLDO is shown in Figure 1. $V_{ref}$ and clk are the inputs, and $V_{out}$ is the output of the DLDO, which is composed of *N* parallel PMOS transistors ($M_{i}$) and a feedback control loop to adjust the output voltage. A shift register is implemented in conventional DLDOs to digitally control the PMOS transistors. The schematic of the shift register used in the design is illustrated in Figure 2, where $Q_{i}$ is the controller output to control the PMOS pass transistors, $V_{cmp}$ is the signal which is the output of the comparator as shown in Figure 1, and Set signal is connected to the ground. A shift register is typically composed of flip flops and logic inputs. A digital controller produces the logic outputs, as illustrated in Figure 3, where $M_{i}$ is the *i*th PMOS, $Q_{i}$ is the logic output of the digital controller, and *i* denotes the activation stage of the digital controller. The shift register is controlled by $V_{cmp}$ at the rising edge of each clock cycle to control the PMOS transistors simultaneously. As shown in Figure 3, $Q_{n+1}$ is turned on (off) when $V_{cmp}$ is high (low) and the shift register shifts right (left) [25].

### 2.2. Countermeasures against Side-Channel Attacks

Countermeasures can be categorized into two based on the implementation. Software countermeasures are designed at the software level typically in a micro-controller. Hardware countermeasures are implemented directly during the design process by modifying the circuits and a dedicated protection circuit is implemented as a countermeasure. There are therefore distinct differences between software and hardware countermeasures. The focus of this paper is primarily on hardware countermeasures; therefore, a brief background of hardware countermeasures is provided below.

Hardware countermeasures can perform both hiding and masking of the private data. The primary hiding countermeasures are decoupling, minimization, randomization, desynchronization, and noise insertion [1]. Shamir first suggested the use of decoupling capacitors to improve the security of a cryptographic circuit which runs operations with sensitive information [28]. A variable capacitor that is embedded into smart cards has been demonstrated to improve the resistance against side-channel attacks for cryptographic circuits [29]. A current source and a decoupling capacitor are embedded into a cryptographic processor to improve side-channel resistance against power side-channel attacks [30]. A current equalizer is proposed in [31], which utilizes switch capacitors to hide the power usage of a cryptographic processor. A current–injection loop is proposed in [32] to remove both the low and high frequency variations in the supply current. A decoupling architecture as a countermeasure which is embedded in the power management system is proposed in [1]. A current flattening technique is proposed in [33], where additional current is injected to mitigate the fluctuations in the current consumption, which makes the power analysis attacks more difficult to succeed. Additionally, different types of on-chip voltage regulators are demonstrated to improve the resistance against power side-channel attacks in [21].

In addition to the aforementioned circuit level countermeasures that specifically target analog circuitry, several other countermeasures modify the digital portion of the circuitry. New logic families are introduced to balance and hide the power consumption of the logic core that implements the encryption circuitry. Sense-amplifier based logic [34], wave dynamic differential logic [35], dual-rail circuits [36], MOS current mode logic [37], and adiabatic and dual rail circuits are among the gate level countermeasures [38] against power and electromagnetic (EM) based side-channel attacks. The power consumption and area overhead of gate level countermeasures are typically high [35]. Additionally, customized libraries are required when the logic style is altered.

There is also a variety of high-level architectural countermeasures that can typically work orthogonal with the countermeasures at different levels of design abstraction, including the circuit based countermeasures. The sensitive information is balanced between multiprocessors with an algorithmic level balancing algorithm to improve resistance against power side-channel attacks in [39], and a reconfigurable hardware methodology is proposed in [40], where reconfigurable functional units are proposed to improve the side-channel resistance against power side-channel attacks for FPGAs. A technique to randomize the time interval in S-box shift operations is proposed against differential power analysis attacks in [41]. The power and area overhead of the hardware countermeasures increase with more levels of design abstraction; however, those countermeasures that cross-cut multiple abstraction levels offer increased protection [1]. The security problems are investigated in the following articles that can be the basis to design a countermeasure. The crosstalk implications of the long wires due to the routing in an FPGA are studied in [10], and were suggested as a possible countermeasure. The information leakage between independent Advanced Encryption Standard (AES) circuit blocks is investigated in [11]. A possible attack method is proposed for independent logic blocks in an FPGA because two applications share the same FPGA resources in [12]. The effect of IR voltage drop and crosstalk due to the inter-wire capacitance, the capacitance between neighboring wires in an integrated circuit, for hardware masking in FPGA is investigated in [13].

Hardware masking is a shuffling type of countermeasure and is the main concern of this paper. The basic developments can be summarized as the hardware masking being designed in FPGA and related security tests being implemented in [5]. The implications of certain power delivery network parameters for the hardware masking are investigated on an ASIC design, and the security benefits of the hardware masking have been demonstrated to alter when these parasitic elements change in [7]. The power delivery network parameters have been further investigated in an ASIC design, and the correlation between masking shares has been shown to be highly related with the power delivery network in [8]. The security metrics based on mutual information, and heuristic tools for hardware masking are developed in [4]. The security implications of the crosstalk in switching CMOS gates for hardware masking are investigated in [9].

### 2.3. Hardware Masking

A hardware masking technique splits the sensitive information to be processed into multiple shares. Shamir’s secret sharing scheme provides an effective way to divide the sensitive information into multiple shares which are processed individually [42]. These shares are assumed to be uniformly distributed and random. One of the methods for hardware masking is Boolean masking, which splits the sensitive information into multiple smaller portions so that the Boolean addition of each share constitutes the sensitive information. In a *d*th order Boolean masking, the sensitive information is divided into d+1 shares where the Boolean addition of individual data utilized in each share produces the sensitive information.

After the proposal of Shamir’s secret sharing scheme, many hardware masking designs have been proposed [5]. Prior work based on Shamir’s secret sharing scheme fails to provide sufficient security because the non-ideal behavior of integrated circuits in practical implementations has not been given sufficient attention. Additionally, there is also a gap between the theory and practical implementation of hardware masking due to the propagation of glitches in the circuit and sequential design approach, which may lead to a certain amount of bias, the violating of the randomness and uniformity [13,43,44]. To prevent the glitches from being propagated between masking shares, two types of hardware masking are proposed, threshold implementation (TI) and domain-oriented masking (DOM) [5].

TI, one of the widely used masking schemes, overcomes the glitch propagation among shares with non-completeness, which means that any multiple component function should be independent of all unshared functions to achieve the security of hardware masking. The design becomes glitch-resistant because the components through which glitch impacts other shares becomes independent. The non-completeness can be formulated for a 2nd degree function with three shares as

(1) $$\begin{array}{c} S(x,y,z)=x+yz\\ S_{1}=x_{2}+y_{2}z_{2}+y_{2}z_{3}+y_{3}z_{2}\\ S_{2}=x_{3}+y_{3}z_{3}+y_{3}z_{1}+y_{1}z_{3}\\ S_{3}=x_{1}+y_{1}z_{1}+y_{1}z_{2}+y_{2}z_{1} \end{array}$$

where the three shares are distributed among the second order functions with independent variables to maintain a sufficient level of security for hardware masking with non-completeness [5].

DOM is another type of hardware masking, which uses operation refreshing and share compression in two clock cycles. In the refreshing operation, the randomness is inserted during the multiplication process. In the share compression, all of the operations in a share are synchronized, and each share is implemented in a dedicated domain. For example, the individual shares of *x* such as $x_{1}$ and $x_{2}$ are assigned to domain one and domain two, respectively. The domains are implemented independently from each other. Therefore, a *d*th order masking is secure against *d*th order attacks as long as each domain is independent. The implementation is glitch resistant since there is no common source between shares where the glitch can propagate. The required number of components is less than that of TI with an additional cost of an extra clock cycle [5].

The independence of hardware masking is just an assumption when theoretically demonstrating the effectiveness of these masking techniques. However, practical designs may suffer due to the difficulty of designing actual circuits that have statistically non-significant coupling (and consequently leakage) between circuit blocks that share the same die [5].

The existing studies [14,15,16] dealing with the security vulnerability of hardware masking only focus on the problems via place and route, importing existing technologies, and 3D CMOS stacking techniques to reduce the leakage within the integrated circuit. Moreover, a limited number of studies focus on the PDN and inserting voltage fluctuations in a secure aware way has not been studied well. The optimization of voltage fluctuations within PDN is studied in [18,19], but the security aspects of voltage fluctuations are not studied in these works. Moreover, to the best of our knowledge, a novel way of using DLDO within PDN to solve the security aspects of hardware masking has not been proposed previously. Therefore, a design space exploration of the implications of different voltage regulator topologies and placement techniques for the voltage regulators and masking shares is performed to demonstrate the effectiveness towards closing the gap between theory and practice for hardware masking implementations.

## 3. Theoretical Modelling

In this paper, a first-order masking scheme is implemented such that the sensitive information is divided into two shares. The presence of noise coupled from neighboring circuitry to the masking shares is crucial to make the simulations more realistic and a better representation of a practical integrated circuit. Accordingly, a Fibonacci linear-feedback shift register (LFSR) is implemented to emulate the noise that stems from another circuitry. A 16-bit LFSR is used with four taps [45,46].

A t-shaped PDN is designed to represent the power delivery from an off-chip voltage regulator to the on-chip masking shares. The two masking shares are expected to exhibit significant noise coupling when connected directly to an external voltage regulator through this shared PDN. Accordingly, any one of the masking shares can potentially leak information to an adversary about the other shares related to the private information. The concurrent operation of the shares makes it slightly more difficult to extract sensitive information from a single share from another share. Additionally, the relative position of the shares with respect to the other shares and voltage regulator connections within the PDN are expected to significantly impact the coupling. A shunt resistor can be connected to the input power line of the circuit by the attacker to measure the power usage of the cryptographic device. The shares are represented with XOR gates as cryptographic circuits commonly utilize XOR gates to perform encryption operation [7].

**Figure 4:**
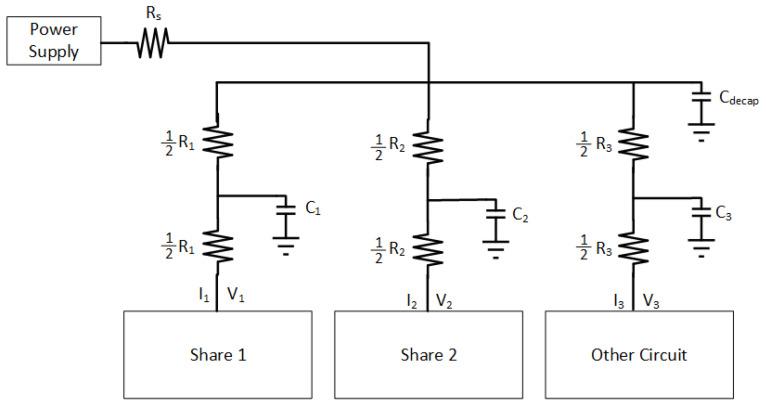
PDN model with masking shares and other circuitry which is modeled with a linear feedback shift register (LFSR).

A basic schematic of the PDN with an off-chip power supply is illustrated in Figure 4. $R_{S}$ represents the shunt resistor that an attacker can connect to collect measurements from the power supply noise. The supply voltage on the local node of Share${}_{1}$ and Share${}_{2}$ are represented, respectively, with $V_{1}$ and $V_{2}$. $I_{1}$ and $I_{2}$ are, respectively, the current provided to Share${}_{1}$ and Share${}_{2}$. $R_{1}$ and $R_{2}$ are the parasitic resistances that basically reduce with closer proximity of the related load circuit to the power supply connection. $C_{decap}$ is the decoupling capacitor that is connected to the off-chip power supply. $C_{1}$ and $C_{2}$ represent the parasitic capacitance of the PDN. $V_{3}$, $I_{3}$, $C_{3}$, and $R_{3}$ are the circuit parameters for the other circuit, which models all of the neighboring circuitry and is represented by an LFSR.

A quantitative analysis of the circuit is performed by deriving the related transfer functions. The I−V relationship is determined using basic circuit theory based on the Figure 4. First, only parasitic elements are considered to find the I−V relationship.

According to Kirchhoff current law, $V_{joint}$ can be written as

(2) $$\frac{V_{dd}-V_{joint}}{R_{s}}=\frac{V_{joint}-V_{1}}{R_{1}}+\frac{V_{joint}-V_{2}}{R_{2}}+I_{other}.$$

With Kirchhoff voltage law,

(3) $$V_{joint}=V_{2}+I_{2}R_{2}.$$

After inserting (3) into (2) and organizing the algebraic expression, the current supplied to Share${}_{2}$ becomes

(4) $$I_{2}=\frac{V_{joint}-V_{2}}{R_{2}}=\frac{V_{dd}R_{1}+V_{1}R_{s}-V_{2}R_{1}-V_{2}R_{s}-I_{other}R_{1}R_{s}}{R_{t}},$$

where $R_{t}=R_{1}R_{2}+R_{1}R_{s}+R_{2}R_{s}$.

The relationship between $I_{1}$ and other components is determined using the Kirchhoff voltage law as

(5) $$I_{1}=\frac{V_{dd}-V_{joint}}{R_{s}}-I_{2}-I_{other}.$$

After inserting (3) into (5), $I_{1}$ can be written as

(6) $$I_{1}=\frac{V_{dd}-\left(Rs+R_{2}\right)I_{2}-I_{other}R_{s}-V_{2}}{R_{s}}.$$

The hardware masking consists of XOR gates and inverters at the last state of the circuit logic. Therefore, the assumption is made that a capacitive load is connected to the output of inverter. The CMOS logic consumes power during the operation of the encryption circuitry while charging the output capacitor during input logic changing from a one to zero state. This current passes through the PMOS transistor. Therefore, the relationship between the activity of the circuitry and current through the PMOS is modeled. The PMOS is assumed to work in the linear region during the switching activity. The modeling is performed according to the CMOS logic and can be applied to any MOSFET technology where the basic MOSFET equations are valid [47]. The second order parameters are neglected to be zero since the operation of PMOS is under the 1 V, and the contribution of second order parameters is low. The resistance of PMOS is assumed to be

(7) $$R_{p}=\frac{1}{\beta_{p}(V_{SG}-|V_{tp}\left|\right)},$$

where $\beta_{p}$ is the coefficient that includes W/L ratio, mobility, gate oxide area, and other parasitic related parameters. The current through PMOS occurs when CMOS input signal changes from one to zero. The current that passes through the PMOS transistor and charges the output capacitance can be written as

(8) $$i_{out}=\frac{V_{supply}}{R_{p}}e^{-\frac{t}{\tau_{p}}},$$

where $\tau_{p}$ is the time constant for the output capacitance and product of $R_{p}$ and output capacitance. After replacing $R_{p}$ with (7), $i_{out}$ can be written as

(9) $$i_{out}=\beta_{p}(V_{SG}-|V_{tp}\left|\right)V_{supply}e^{-\frac{t}{\tau_{p}}}.$$

The time interval is selected when the maximum voltage drop occurs, leading to maximum current in PMOS. At this time interval, $V_{SG}$ is equal to $V_{supply}$. Thus, $i_{out}$ becomes

(10) $$i_{out}=\beta_{p}(V_{supply}-|V_{tp}\left|\right)V_{supply}.$$

$i_{out}$ can be expanded into total current in the circuit. In this case,

(11) $$i_{total}=\sum_{i=1}^{n}\alpha_{i}\beta_{pi}(V_{supply}-|V_{tp}\left|\right)V_{supply},$$

where $\beta_{pi}$ is the $\beta_{p}$ for each PMOS, and $\alpha_{i}$ is the number of PMOS for each *i*. Further simplification can be performed for the summation of $\alpha_{i}\beta_{i}$ replaced by α. α represents the fraction of the data being processes in a clock cycle. $i_{total}$ becomes

(12) $$i_{total}=\alpha (V_{supply}-|V_{tp}\left|\right)V_{supply}.$$

The current in Share${}_{1}$ can be defined using (12) as

(13) $$I_{1}=\alpha_{1}V_{1}(V_{1}-|V_{tp}\left|\right),$$

where $\alpha_{1}$ is the fraction of the data being processed in a clock cycle for the Share${}_{1}$.

(4), (6), and (13) yield to $V_{2}$ as

(14) $$\begin{array}{c} V_{2}=\frac{\alpha_{1}R_{t}}{R_{s}}V_{1}^{2}+\frac{R_{s}+R_{2}-\alpha_{1}R_{t}\left|V_{tp}\right|}{R_{s}}V_{1}-\frac{R_{2}}{R_{s}}V_{dd}+I_{other}R_{2}, \end{array}$$

where $R_{t}=R_{1}R_{2}+R_{1}R_{s}+R_{2}R_{s}$.

A similar analysis is performed with the decoupling capacitor with Kirchhoff current law yielding

(15) $$\begin{array}{c} \frac{V_{dd}-V_{joint}}{R_{s}}=\frac{V_{joint}-\left(V_{1}+\frac{1}{2}I_{1}R_{1}\right)}{\frac{1}{2}R_{1}}+\frac{V_{joint}-\left(V_{2}+\frac{1}{2}I_{2}R_{2}\right)}{\frac{1}{2}R_{2}}+\\ \frac{V_{joint}-\left(V_{3}+\frac{1}{2}I_{other}R_{3}\right)}{\frac{1}{2}R_{3}}+C_{decap}\frac{dV_{joint}}{dt}. \end{array}$$

With Kirchhoff voltage law

(16) $$\begin{array}{c} V_{joint}=\frac{1}{2}I_{1}R_{1}+V_{1}+\frac{1}{2}R_{1}\left(I_{1}+\frac{C_{1}d\left(\frac{1}{2}I_{1}R_{1}+V_{1}\right)}{dt}\right). \end{array}$$

(15) and (16) yield

(17) $$\begin{array}{c} V_{2}=\frac{1}{2}R_{s}C_{decap}\left(\frac{dV_{1}}{dt}+\frac{1}{2}R_{1}C_{1}\frac{d^{2}V_{1}}{dt^{2}}\right)\\ +\frac{1}{2}C_{1}R_{1}\frac{dV_{1}}{dt}\left(\frac{R_{2}}{R_{2}}+1+\frac{R_{2}}{R_{3}}+\frac{R_{2}}{2R_{s}}\right)+V_{1}\left(1+\frac{R_{2}}{R_{3}}+\frac{R_{2}}{2R_{s}}\right)\\ -\frac{R_{2}}{R_{3}}V_{3}-\frac{1}{2}I_{other}R_{2}-\frac{R_{2}}{2R_{s}}V_{dd}. \end{array}$$

**Figure 5:**
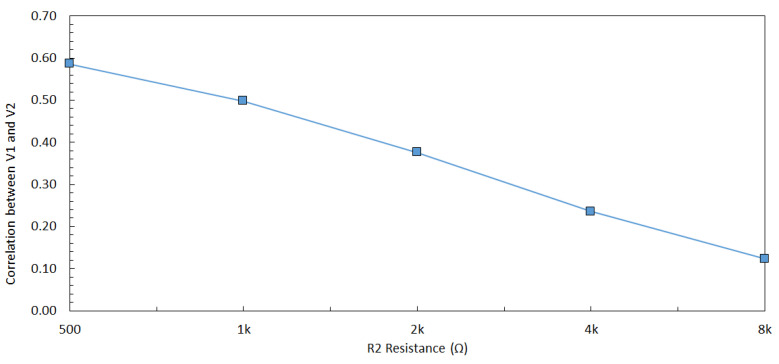
Correlation between $V_{1}$ and $V_{2}$ with 100,000 traces when $R_{2}$ is changed from 500 Ω and 8 kΩ.

The relationship between the supply voltage values delivered to Share${}_{1}$ and Share${}_{2}$ can be observed in (14) and (17). The methodology of our experiments explained in the next section are based on the dependency of the delivered supply voltages to the shares. The aim of the theoretical analysis is to demonstrate the direct relationship between the input voltages of the shares ($V_{1}$ and $V_{2}$) and $R_{2}$. The experimental simulations are done with 32 nm PTM [48], and the direct relationship between $V_{1}$, $V_{2}$, and $R_{2}$ can be observed in Figure 5 where this relationship can be observed (15) and (16). Moreover, a complementary simulation is made with Cadence Virtuoso based on a 28 nm FDSOI CMOS technology and the similar relationship in Figure 5 is observed with 300 traces. However, the simulations cannot go beyond 300 traces because of the limitations of Cadence Virtuoso simulator; therefore, comprehensive simulations are made with 32 nm PTM using the Synopsys Finesim. (15) and (16) can be applied to any kind of hardware masking implementation and independent of an encryption algorithm where hardware masking is applicable.

## 4. Methodology

The parasitic impedance can be partially reduced using advanced placement and routing algorithms, and the design process requires iterative methods to minimize the effect of the parasitic impedance. Although the parasitic impedances cannot be eliminated completely, there are various techniques to minimize the detrimental effects of the interconnect parasitics on the system performance [49]. The parasitic elements and parasitic impedance can be modeled with the help of several design automation tools. The time dependent voltage fluctuations as a result of these parasitic impedances cause the primary coupling mechanism among masking shares through the shared PDN. The power supply noise can be partially mitigated with a careful modification of the PDN and placement of on-chip voltage regulators. Therefore, in this paper, a design space exploration of DLDO voltage regulators and PDN for hardware masking is performed to minimize the coupling among masking shares through the shared PDN, partially closing the gap between the theory and practice for hardware masking.

Since several hundreds of thousands of simulations under different inputs and variations need to be performed to obtain meaningful results in the proposed statistical tests, a drastically improved simulation speed and capacity are required. Accordingly, Synopsys Finesim, a SPICE circuit simulator, is used throughout the paper to improve the simulation time. In addition, 32 nm PTM CMOS technology models have been used [48]. The supply voltage is set to 1 V. Each share is represented as logic gates to emulate the cryptographic operation, similar to [7,14].

**Figure 6:**
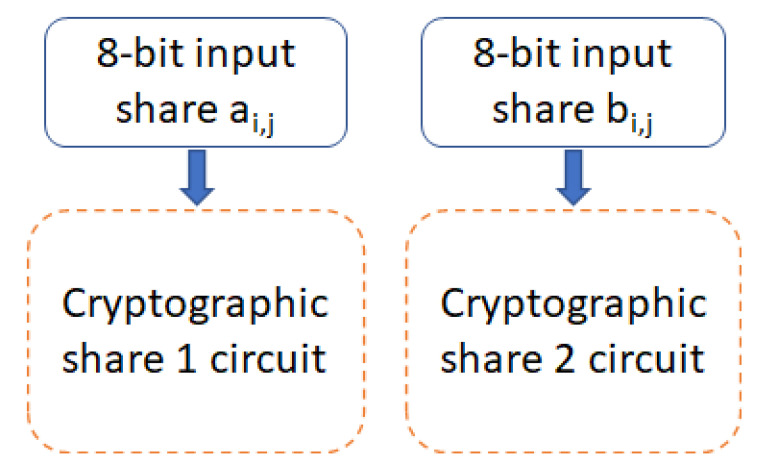
8-bit input a${}_{i,j}$ for Share${}_{1}$ and 8-bit input b${}_{i,j}$ for Share${}_{2}$, where *i* is the input bit, and *j* is the share number.

The Boolean function of $GF(2^{n})$ is used to emulate a cryptographic circuit. A two input XOR gate is utilized as the target circuit [7]. A Boolean masking scheme is utilized, which uses TI. The 8-bit input is divided into two shares using XOR gates, as illustrated in Figure 6. $a_{i,j}$ stands for the first input vector of the share, and $b_{i,j}$ stands for the second input vector, where *i* is the input size, i=1,2,3,4,5,6,7,8, and *j* is the share number, j=1,2. This circuit is theoretically secure against first-order side-channel attacks. A small sized circuitry is chosen specifically to further speed up the SPICE simulations. $2^{8\times 2}-2^{8}$ nontrivial input changes are created to emulate all input changes in the simulations, and the XOR circuits for masking shares are adapted from [7].

In this article, the correlation between the node voltages $V_{1}$ and $V_{2}$ is investigated in order to assess the noise coupling between either from Share${}_{1}$ to Share${}_{2}$ or from Share${}_{2}$ to Share${}_{1}$. The correlation between $V_{1}$ and $V_{2}$ is a strong indication of a possible violation of the independence assumption of the masking shares. If the correlation is zero, the shares are uncorrelated and do not affect each other, leading to a potentially effective hardware masking implementation. Alternatively, if the correlation is closer to the maximum value of one, the shares have significant impact on the other shares, leading to a poor hardware masking implementation. In addition to correlation, Welch’s *t*-test is applied to the proposed hardware masking design. Welch’s *t*-test is widely used to quantify security where the level of sensitive information leakage can be observed quantitatively. Typically, a *t*-test value of 4.5 and below is assumed to be secure since the amount of information leakage from one share to another share is considered negligible [50].

Welch’s *t*-test is used to check if the circuit behaves differently under two different inputs, e.g., one is fixed vs. one is random, and can be written as

(18) $$\begin{array}{c} t\left(X,Y\right)=\frac{E(X)-E(Y)}{\sqrt{\frac{\sigma_{X}^{2}}{N_{X}}+\frac{\sigma_{Y}^{2}}{N_{Y}}}}, \end{array}$$

where *X* and *Y* are two random distributions, *E*(*X*) and *E*(*Y*) are the expected value of *X* and *Y*, and $\sigma_{X}$ and $\sigma_{Y}$ are the standard deviation. The hypothesis testing methodology is used to determine the resemblance of *X* and *Y*. If *t*(*X*,*Y*) is lower than 4.5, the confidence interval of the test is 99.99%, meaning that *X* is statistically different than *Y*. Therefore, the *t*-test values below 4.5 are typically assumed to have no leakage [5,7,14].

Voltage fluctuations in the power delivery network (that are highly correlated with the switching activity of the individual masking shares) are the primary source of coupling between hardware masking shares. Therefore, voltage fluctuations are generally analyzed to measure the leakage. One of the sources of voltage fluctuations is the change in the current demand over time due to the switching activity of the circuits which are powered through a PDN that is comprised of a resistive and capacitive interconnection network. The voltage fluctuations can be as fast as the operating frequency of the load circuit, leading to considerable voltage fluctuations when the switching activity is higher.

**Figure 7:**
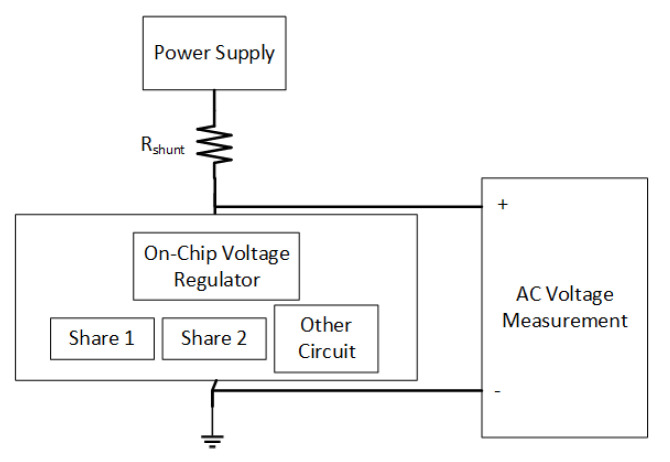
Experimental setup used for the *t*-test is shown where $R_{shunt}$ is the shunt resistor.

**Figure 8:**
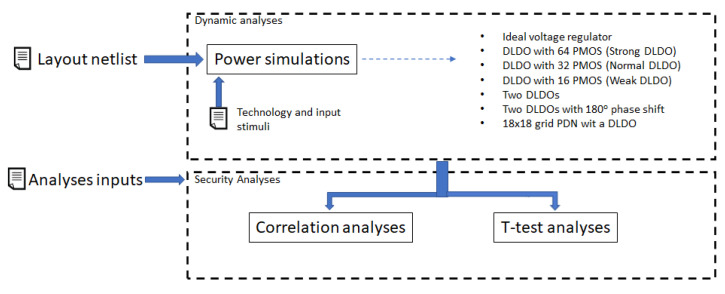
Flowchart of the procedure of experiments and security analyses.

Throughout this paper, the voltage fluctuations are used for evaluation by utilizing the previously known methods of side channel analysis, such as Welch’s *t*-test and correlation. The leakage testing methodology is defined in [50]. However, the experimental method used in this paper needs to be detailed, as explained below. The circuit is assumed to run the hardware masking with two shares. Placing a shunt resistor in main power line is a common practice in side channel analysis [50]. The voltage fluctuations are therefore assumed to be measured with the help of the $R_{shunt}$ resistor. The changes in the supply current generate voltage fluctuations over the shunt resistor. $R_{shunt}$ resistor is selected as 1 Ω. The evaluation is performed for different situations; with an ideal voltage regulator, with a DLDO implemented at the transistor level, with a simple PDN, and with a more realistic PDN implemented as a grid. The placement of on-chip voltage regulator is described in the experimental results. A fixed vs. random *t*-test is performed. The experiment setup is shown in Figure 7, and flowchart of the experimentation is summarized in Figure 8.

The source code is available at https://github.com/sonersec/Exploiting-On-chip-Voltage-Regulators-for-Leakage-Reduction-in-Hardware-Masking, accessed on 16 August 2022.

## 5. Verification and Validation of Inputs

**Figure 9:**
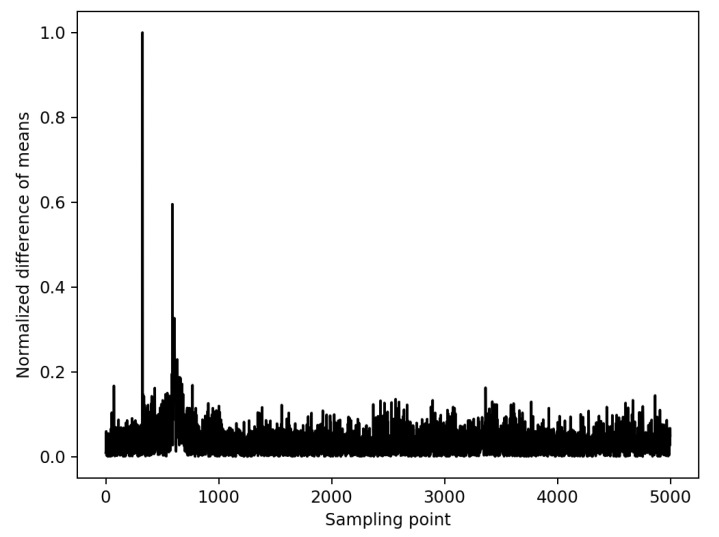
Differential power analysis on AES 128-bit with 100,000 traces on Atmega128 8-bit AVR.

**Figure 10:**
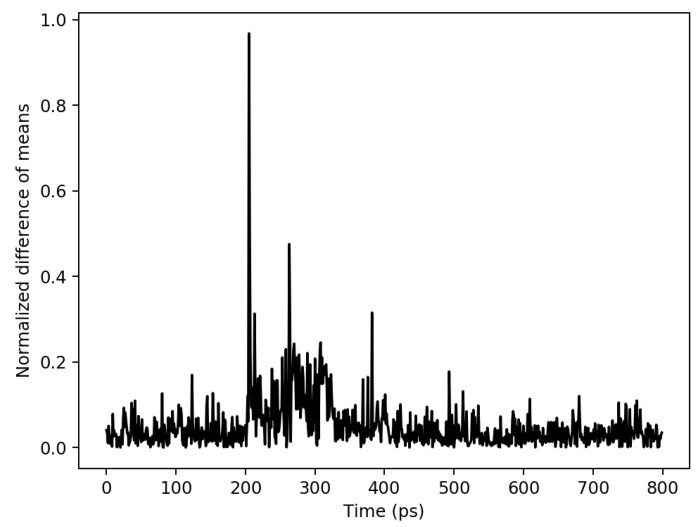
Differential power analysis on ASIC design with 100,000 traces.

To validate the inputs that are used in the experiments, a side-channel attack is performed on a real encryption device when processing the same inputs. A 128-bit AES is run on an Atmega128 8-bit AVR device. In addition, 100,000 traces are collected with Chipwhisperer [51]. The inputs that are used in the experiments are used as an input in the encryption device. These inputs are completed to 128-bit by replication since each S-box in AES is 8-bit, and there are 16 S-box units. Differential power analysis is a side channel attack and widely used to extract the correct key from the encryption devices [52]. Therefore, a differential power analysis is implemented on the real encryption device with the generated inputs in the experiments. The difference of means peaks at 365th sampling point when the correct key is found as shown in Figure 9. The same attack is performed on the proposed ASIC design with extensive simulations where the same inputs are used. The results are shown in Figure 10 as the difference of means peaks at 205 ps when the correct key is found. The same inputs are tested both in the proposed ASIC simulations and real encryption device, and the correct key is extracted in both of the attacks; validating the simulation inputs is sufficiently good to represent practical inputs. To verify the inputs, the theoretical model and experimental results should complement each other. Therefore, an experiment is implemented as discussed in Section 6. There is a relationship between $V_{1}$ and $V_{2}$ as shown in Figure 5 when $R_{2}$ is changed from 500 Ω to 8 kΩ. This relationship complies with the analogy that, when $R_{2}$ increases, the correlation decreases as $R_{2}$ is related with the physical distance between two hardware masking shares.

## 6. Experimental Results

**Figure 11:**
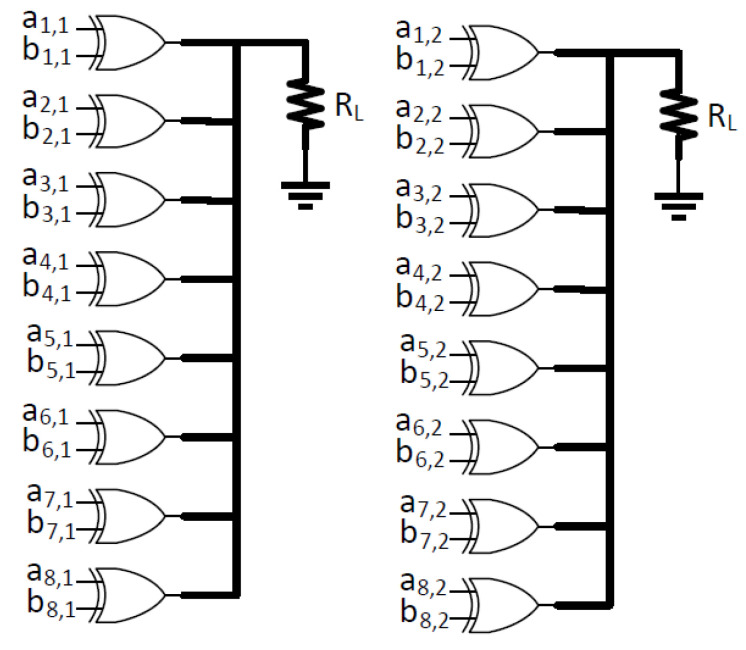
8-bit two-share XOR with $a_{i,j}$ and $b_{i,j}$ for $Share_{1}$ and $Share_{2}$, where *i* is the input bit, *j* is the share number, and $R_{L}$ is 100 Ω.

**Figure 12:**
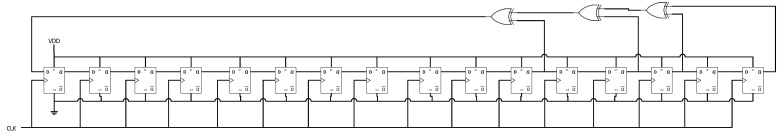
16-bit Fibonacci LFSR where CLK is the clock signal.

A thorough analysis to evaluate the effects of the PDN parasitic impedance and different on-chip voltage regulator connection strategies is performed in this section. The target circuit consists of XOR gates with two masking shares. The simulations are performed using Synopsys Finesim, and the results are used in correlation analysis and *t*-test evaluation. In the experiments, interconnect parasitic impedances are assumed as $R_{s}=1$ kΩ, $C_{1}=C_{2}=C_{3}=1fF$, $R_{2}=2k$ and $R_{3}=1\;\Omega$. A schematic of the PDN and related circuitry used in the simulations are depicted in Figure 4. DLDO runs at 5 GHz, and the hardware masking shares run at 1 Ghz in all experiments. A fixed vs. random *t*-test is implemented throughout the experiments [5]. The load circuit for the two-share hardware masking is an XOR gate, as shown in Figure 11, and this XOR load circuit is adapted from [7]. The 16-bit Fibonacci LFSR is shown in Figure 12, which runs at 1 Ghz.

### 6.1. Effect of the PDN Parasitic Impedance with an Ideal Voltage Regulator

The effect of the PDN parasitic impedance is investigated under fixed input to Share${}_{1}$ and random input to Share${}_{2}$, which is defined as a fixed vs. random *t*-test [5]. The random input causes fluctuations on the power supply voltage, and these fluctuations propagate within the PDN, affecting the circuitry that is connected to the same PDN. When the circuit blocks are physically closer to each other, the effective PDN impedance between circuit blocks is reduced and the impact of power noise coupling becomes more prominent. In addition, 100,000 traces are collected during each simulation to evaluate the noise coupling from Share${}_{1}$ to Share${}_{2}$.

**Figure 13:**
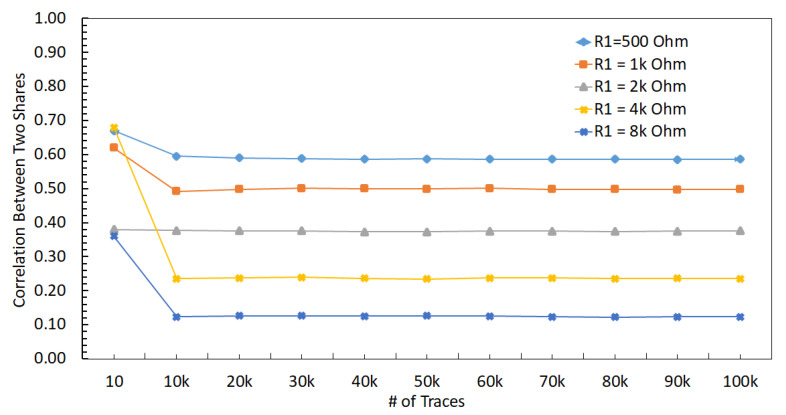
Correlation between $V_{1}$ and $V_{2}$ when R1 is changed from from 500 Ω to 8 kΩ, and voltage supply is ideal.

**Figure 14:**
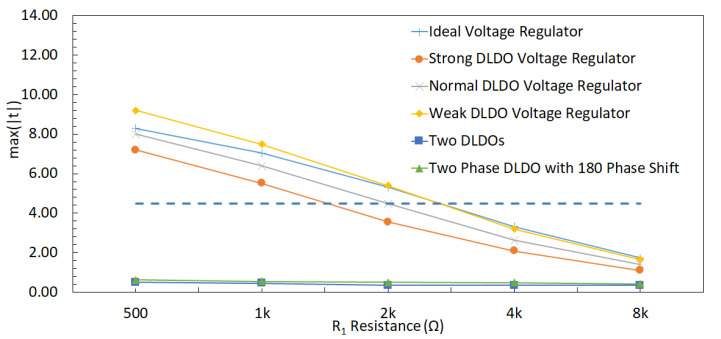
Result of the *t*-test to evaluate the leakage amount between masking shares with different on-chip voltage regulators when the R1 is changed from 500 Ω to 8 kΩ with 100,000 traces.

The relationship between $V_{1}$ and $V_{2}$ can be observed in (3), where an increase in $R_{1}$ or $R_{2}$ reduces the effect of $V_{1}$ on $V_{2}$, i.e., reduces the correlation between $V_{1}$ and $V_{2}$. The correlation between $V_{1}$ and $V_{2}$ decreases when $R_{1}$ changes from 500 Ω to 8 kΩ, which can be observed in Figure 13. The correlation between the two shares decreases when $R_{1}$ increases because the effect of other share decreases (i.e., the relative distance between shares increases). A similar trend with the correlation analysis can be observed in the *t*-test results, as shown in Figure 14, where an increase in $R_{1}$ decreases the *max*(|t|), leading to a more secure design.

### 6.2. Effect of the PDN Parasitic Impedance with a DLDO Voltage Regulator

**Figure 15:**
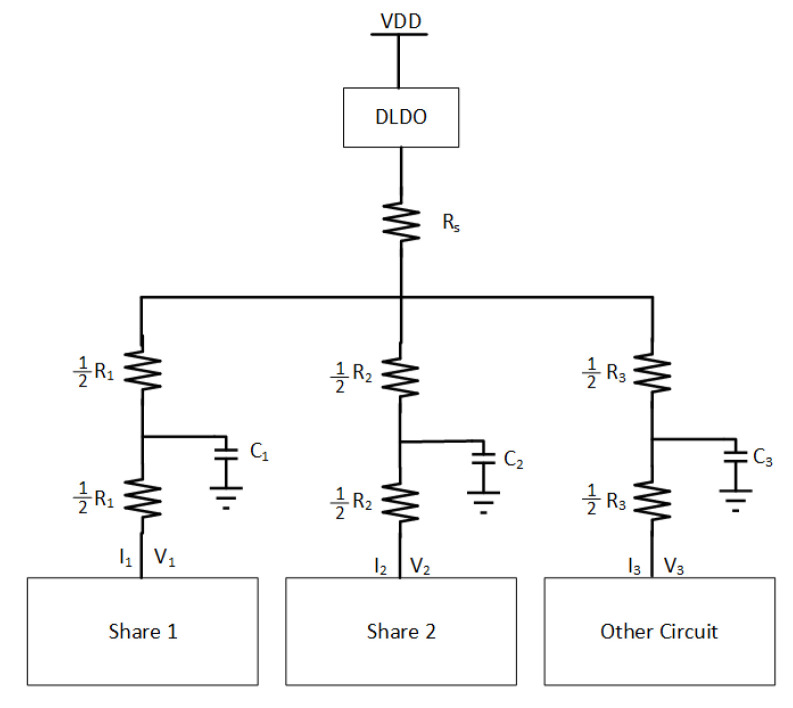
PDN, masking shares, and other circuitry with a single DLDO.

The implications of using a DLDO voltage regulator instead of an ideal supply voltage on the noise coupling from Share${}_{1}$ to Share${}_{2}$ are investigated in this section under different PDN parasitic impedances. Note that the PDN parasitic impedance models both the physical characteristics of the PDN and physical placement of the circuit blocks and voltage regulator within an integrated circuit. The PDN parasitics include the impedance of the interconnect wires, capacitive coupling among neighboring interconnects, and parasitic impedance of the load circuitry. For example, the idle circuitry can be modeled with a lumped capacitor, whose value depends on the total gate capacitance of the load circuitry. The implications of the parasitic impedances are investigated in this section and the security evaluation is performed with a *t*-test. A detailed schematic of the simulation is shown in Figure 15. Three different DLDO voltage regulators (i.e., strong (large), normal, and weak (small)) are utilized to better evaluate the impact of the current driving capability of the voltage regulator on the noise coupling between masking shares.

#### 6.2.1. DLDO with 64 PMOS (Strong DLDO)

**Figure 16:**
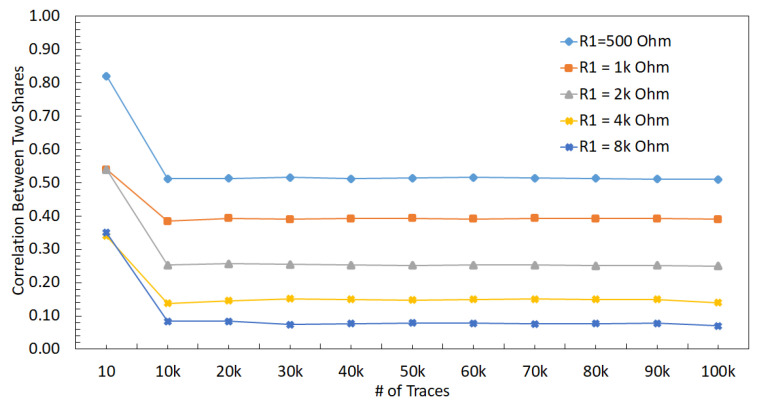
Correlation between $V_{1}$ and $V_{2}$ when R1 is changed from 500 Ω to 8 KΩ when a strong DLDO is used as the voltage regulator.

A similar PDN parasitic impedance evaluation is performed with a strong DLDO voltage regulator instead of an ideal voltage regulator used in the previous section. There are 64 PMOS transistors as the pass transistors within the strong DLDO. The correlation between $V_{1}$ and $V_{2}$ decreases when $R_{1}$ increases, as shown in Figure 16. Additionally, the correlation is lower as compared to the case with the ideal voltage regulator since DLDO inserts a certain amount of noise while regulating the voltage. Furthermore, max(|t|) decreases when $R_{1}$ increases, as shown in Figure 14. As compared to the analysis when the voltage regulator is ideal, the maximum *t*-test value decreases. The *t*-test value is at a minimum when $R_{1}$ increases to 8 kΩ. Since the increase in the values of $R_{1}$ and $R_{2}$ corresponds to an increase in the physical distance between the two shares, the increase in the physical distance between two shares improves the security since correlation and *t*-test decrease. The output voltage of a DLDO has voltage fluctuations depending on the load current characteristics as compared to an ideal voltage regulator which does not have any voltage fluctuations at the output. These voltage fluctuations (i.e., power noise) due to the non-ideality of the DLDO has a positive impact on the security since *t*-test and correlation decrease as compared to the case when the voltage regulator is ideal [53].

#### 6.2.2. DLDO with 32 PMOS (Normal DLDO)

**Figure 17:**
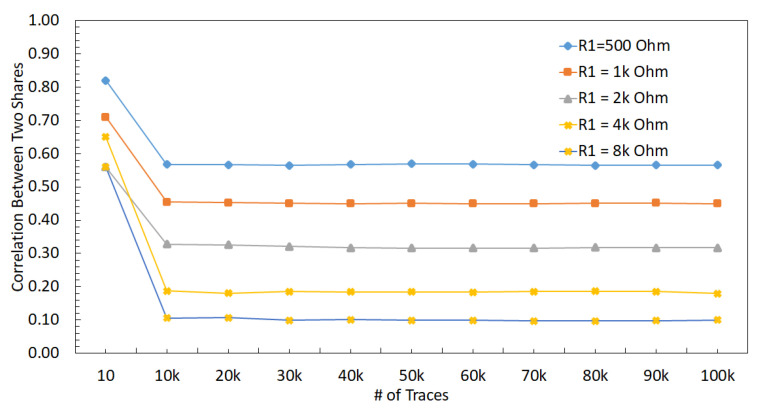
Correlation between $V_{1}$ and $V_{2}$ when R1 is changed from 500 Ω to 8 kΩ when the a normal sized DLDO is used as the voltage regulator.

To evaluate the effect of the size of the voltage regulators, a comparably smaller sized DLDO, which is called normal DLDO, is investigated in this section. The value of $R_{1}$ is changed from 500 Ω to 8 kΩ with a normal DLDO voltage regulator and correlation results between $V_{1}$ and $V_{2}$ are shown in Figure 17. A decrease in the *t*-test value is observed when $R_{1}$ increases, as shown in Figure 14. Since the normal DLDO is weaker than the strong DLDO, the amplitude of the voltage fluctuations increases (i.e., higher power noise) in the output of the DLDO, leading to higher *t*-test values than the case when the voltage regulator is strong DLDO. An increase in the value of $R_{1}$ decreases the correlation between masking shares, leading to a lower *t*-test value, which means the circuit becomes more resistant against side-channel attacks.

#### 6.2.3. DLDO with 16 PMOS (Weak DLDO)

**Figure 18:**
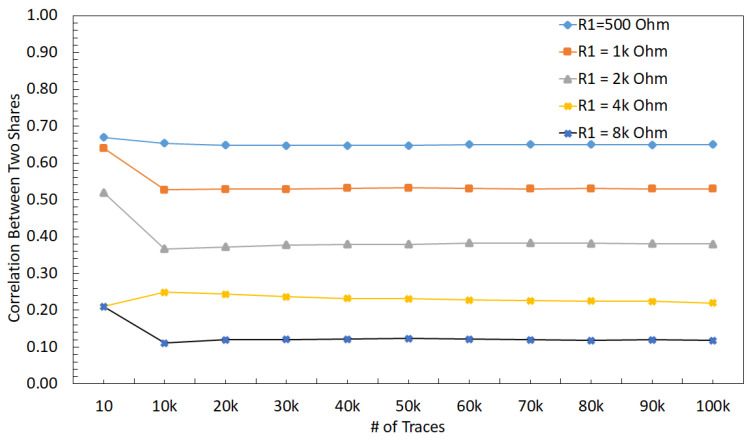
Correlation between $V_{1}$ and $V_{2}$ when R1 is changed from 500 Ω to 8 kΩ when a weak (smaller) DLDO is used as the voltage regulator.

To further investigate the effect of the size of the voltage regulators, an even smaller sized DLDO, which is called weak DLDO, is investigated in this section. The value of $R_{1}$ is changed from 500 Ω to 8 kΩ with a weak DLDO voltage regulator and correlation results between $V_{1}$ and $V_{2}$ are shown in Figure 18. A decrease in the *t*-test value is observed when $R_{1}$ increases as shown in Figure 14. Additionally, the *t*-test values are higher for the weak DLDO than the strong and normal DLDO configurations, since the weak DLDO is smaller than the strong and normal DLDOs, there is more voltage fluctuations (i.e., higher power noise) in the output of the DLDO, leading to higher *t*-test values than the case when the voltage regulator is larger. An increase in the value of $R_{1}$ decreases the correlation between masking shares, leading to a lower *t*-test.

### 6.3. Effect of PDN Parasitic Impedance with Two DLDOs

**Figure 19:**
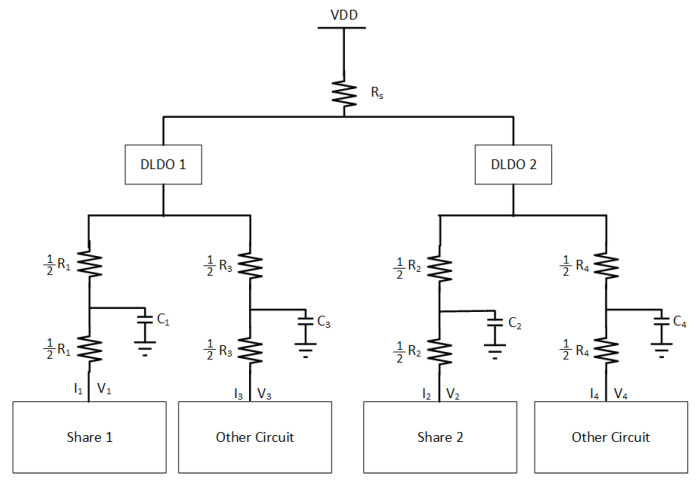
PDN for masking shares and other circuits when each masking share is connected to a dedicated DLDO voltage regulator. This type of connection makes the noise coupling from one share to another significantly more difficult.

The implications of using two separate DLDO voltage regulators that provide power individually to two masking shares on the noise coupling mechanisms is investigated. Note that the DLDO voltage regulators are assumed to be integrated on-chip and their input sides are connected to the same external power supply. The information leakage from one masking share to another share becomes more difficult when the noise has to propagate through a higher number of circuit components and longer interconnect lengths. To simulate the effect of off-chip parasitic impedance, a common $R_{s}$ = 1 kΩ resistor is included. The PDN is shown in Figure 19. $R_{1}$, $R_{2}$, $R_{3}$, $R_{4}$, $C_{1}$, $C_{2}$, $C_{3}$, and $C_{4}$ are the parasitic elements from PDN which connect each share and other circuits to the PDN. Similar to the previous analysis, the other circuit is an LFSR that produces noise by emulating other switching circuitry that is powered by the same on-chip voltage regulator with a masking share. Since, in this analysis, the masking shares are powered by individual DLDOs, an LFSR circuit is connected to each DLDO separately. All of the *t*-test values remain under 4.5, which is assumed to be secure [7] and implies that this configuration provides acceptable security by mitigating the leakage between shares, as shown in Figure 14. All of the values of $R_{1}$ from 500 Ω to 8 kΩ lead to a low correlation between $V_{1}$ and $V_{2}$. The *t*-test value remains constant when $R_{1}$ increases because utilizing individual DLDO voltage regulators keeps the voltage fluctuations low in the shares and provides almost independent operation of two shares.

### 6.4. Effect of PDN Parasitic Impedance with a Shared Two-Phase DLDO

**Figure 20:**
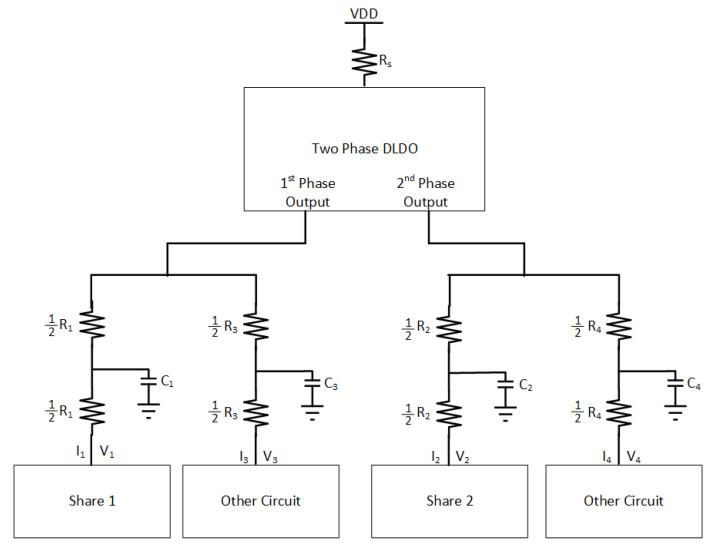
PDN for masking shares and other circuits when each masking share is connected to one of the phases of a two-phase DLDO voltage regulator.

The implications of using a shared DLDO to provide power to two masking shares is investigated. In this case, each share is connected to one of the two phases of the DLDO (i.e., the pass transistors that are controlled by one of the clock signals are connected to one of the shares, the remaining pass transistors that are controlled by the 180${}^{\circ }$ phase shifted version of the clock signal are connected to the second share). Alternatively, half of the pass transistors are driven with same clock and the other half with a $180^{\circ }$ phase shifted clock signal. The basic schematic of the PDN with the two-phase DLDO is shown in Figure 20. $R_{1}$, $R_{2}$, $R_{3}$, $R_{4}$, $C_{1}$, $C_{2}$, $C_{3}$, and $C_{4}$ are the parasitic resistance and capacitance values of the PDN which connect each share and other circuitry to the voltage regulator. Two LFSR circuits are also utilized to be powered separately either with Share${}_{1}$ and Share${}_{2}$.

The *t*-test result for a shared two-phase DLDO is shown in Figure 14. Noise coupling between the shares is higher as compared to the noise coupling using two separate DLDO regulators, leading to an increase in the *t*-test results. The two phases of the DLDO are driven by clock signals which are 180${}^{\circ }$ apart from each other. This phase difference is translated into a slight shift in the voltage fluctuations at the output of these two DLDO phases. This shift inserted by the different clock phases of two-phase DLDO reduces the *t*-test results below the threshold value of 4.5. The *t*-test value remains constant when $R_{1}$ increases because two-phase DLDO keeps the voltage fluctuations low in the shares and minimizes the coupling between the two shares.

The summary of the implications of different PDN design choices on noise coupling is tabulated in Table 1. The minimum noise coupling between the masking shares is achieved with an ideal voltage regulator, which is actually not a practical case, and maximum coupling occurs when the shares are physically close to each other and away from the voltage regulator connections. The minimum coupling in a practical setting can be achieved when each share is powered with a dedicated voltage regulator. Powering each share with a different phase of the voltage regulator provides a trade-off between the design overhead (i.e., area, power consumption, and design difficulty) and security. The correlation decreases with the increase in the number of PMOS in the DLDO and $R_{1}$. The correlation is close to zero with two DLDOs and two-phase DLDO with a 180${}^{\circ }$ phase shift. If the correlation is higher between shares, the hardware masking is less secure than the low correlation case, as can be seen in Figure 14 where *t*-test results are compatible with correlation analyses.

**Figure 21:**
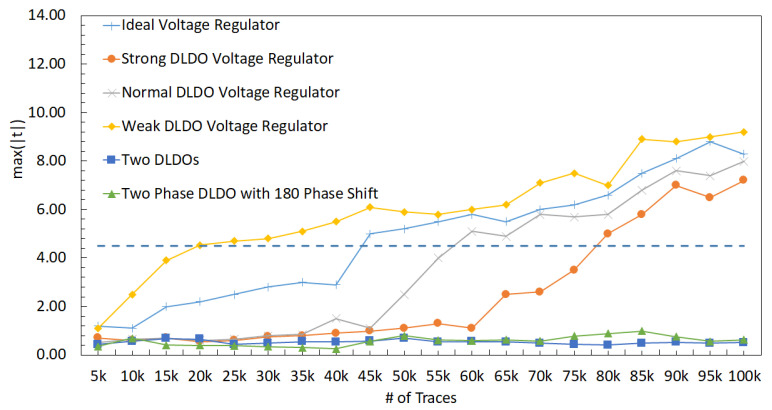
*t*-test value vs. number of traces when $R_{1}$ is 500 Ω.

The leakage occurs within the hardware masking typically after one million traces for FPGAs [5] using first-order t-statistics. However, ref. [5] also reports that the leakage occurs after a couple of thousand traces using second-order leakage analysis. Sijacic et al. discuss in [7] that the leakage starts to become meaningful after nearly 1000 traces for hardware masking with a power delivery network similar to the one used in this work. In our experiments, the leakage from the masking shares occurs after the number of traces is around 20 k when the voltage regulator is a weak DLDO and $R_{1}$ is 500 Ω, as shown in Figure 21. Please note that this is the worst case scenario as the other configurations with different $R_{1}$ values have slightly lower *t*-test values. The leakage does not occur even with 100k traces when either two DLDOs or a two-phase DLDO with phase shift is utilized. The comparison of the other methods is given in Table 2 where *X* means that there is no corresponding results published in the work. The implementation of this work focuses on PDN on ASIC, thus the focus of comparison of this table is based on the PDN.

### 6.5. Effect of the DLDO Voltage Regulator Placement in the Grid Network

**Figure 22:**
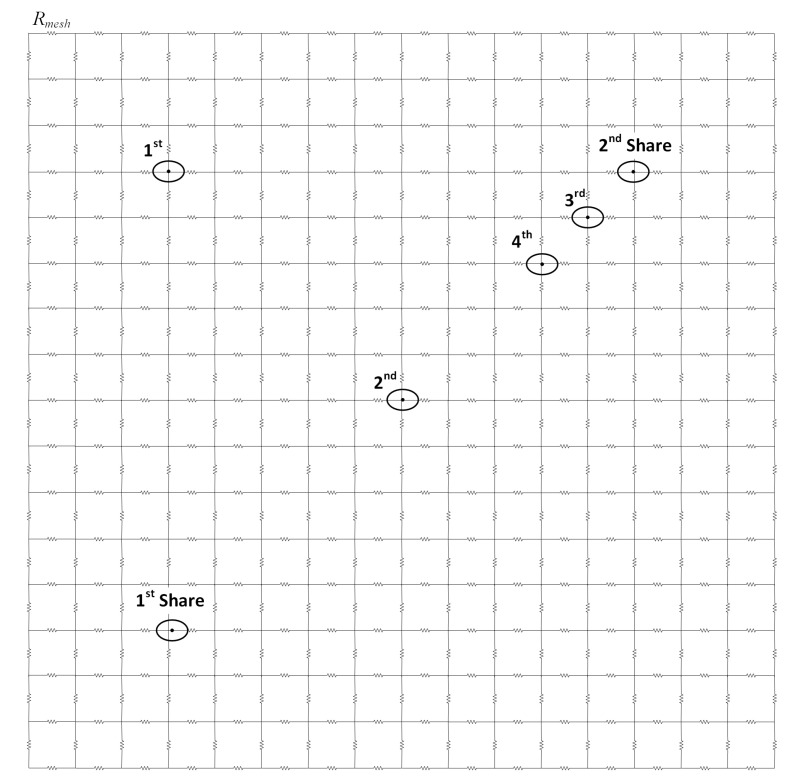
PDN illustrating the placement of 1st and 2nd masking shares and a single DLDO voltage regulator on one of the nodes 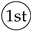, 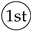, 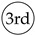, or 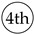.

The design of PDN has a significant impact on the security of the hardware masking, as discussed in the previous sections. To better analyze the implications of PDN and placement of the voltage regulator, a more realistic, 16 × 16 grid structure is investigated, as shown in Figure 22. The effect of the placement of a single DLDO voltage regulator at one of the nodes 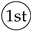, 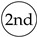, 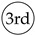, or 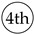, as shown in Figure 22, is investigated where each resistor element of the grid network is 100 Ω. In addition, 100,000 simulations with Finesim are completed for each case when a single DLDO is connected to one of the nodes. The input of the Share${}_{1}$ is constant, and the input of the Share${}_{2}$ is random. The optimal position of DLDO voltage regulator is determined to be 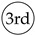, as tabulated in Table 3 because the correlation between the two shares is the lowest as compared to the other cases where the single DLDO is connected to one of the other nodes. The *t*-test value increases as the location of the DLDO is shifted to the far corner of the grid (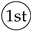). Alternatively, the *t*-test value decreases as the DLDO connection location is shifted to the closest location to the Share${}_{2}$.

The highest *t*-test value is observed when DLDO is located in 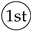; as this is the farthest location from Share${}_{1}$ and Share${}_{2}$. Therefore, based on this observation, the effective resistance in the PDN corresponding to $R_{s}$ is estimated to be high. Similarly, when DLDO is in location 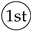, the effective resistance in the PDN corresponding to $R_{s}$ is estimated to be low. Comparing when DLDO is in location 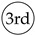; and 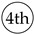, the effective resistance in the PDN corresponding to $R_{1}$ and $R_{2}$ can be estimated. For example, correlation and *t*-test values are lower when the DLDO is in 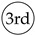; than when the DLDO is in 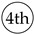;. Thus, the effective resistance between the two shares is lower when DLDO is connected to 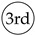; than when DLDO is connected to 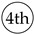;. The voltage drop in Share${}_{1}$ (Share${}_{2}$) affects the voltage drop in Share${}_{2}$ (Share${}_{1}$) more when the effective resistance becomes lower between the two shares. In other words, the security vulnerability of hardware masking due to noise coupling increases when the effective resistance between two shares decreases. As compared to the cases where $R_{1}$ and $R_{2}$ sweep between 500 Ω to 8 kΩ, the *t*-test values decrease with the increase in the distance between shares similar to the case when *t*-test values decrease with the increase in $R_{1}$ in previous experiments. Therefore, the analogy that the increase in the distance improves security holds in the experimental results. The limitation of this work is that the computation times are too high, and large amount of data is created. Parallel and efficient framework is needed to improve the computation times.

## 7. Conclusions and Future Recommendations

Hardware masking relies on the principal assumption that the masking shares are independent of each other. However, this independence assumption can be violated in practical implementations due to the parasitic impedance of on-chip interconnect and circuitry, and coupling between shares. Therefore, a feasible solution to this security vulnerability of hardware masking is proposed in this article. Five different DLDO configurations, strong DLDO, normal DLDO, weak DLDO, two individual DLDOs, and a single two-phase DLDO, are investigated to explore the security implications for hardware masking. Additionally, the noise coupling implications of the DLDO placement are explored when a single DLDO is connected to different locations in the PDN. The correlation between two masking shares and Welch’s *t*-test are used to quantify the amount of noise coupling in hardware masking. The correlation between two masking shares reduces by 80% when a strong DLDO with R1 = 8 kΩ is used. The *t*-test value remains below 4.5 when either two DLDOs separately provide power to the masking shares or different phases of a single DLDO provide power to the masking shares. The optimal placement of a single DLDO voltage regulator among the simulated nodes is determined which reduces correlation between two shares and *t*-test value. Accordingly, design guidelines are proposed that would minimize the gap between the theory and practical implementation of hardware masking.

The amount of data and simulation times are huge. Therefore, the fast simulators should be utilized due to the limitations of the computing resources and timing limitations. A fast simulator with more advanced technologies can be utilized and implemented. The effect of 3D integration with advanced technologies can be adapted to improve the security of the hardware masking.

## Figures and Tables

**Table 1:** Summary of the minimum information leakage results evaluated by a *t*-test for different voltage regulator and PDN design choices.

Setup	$R_{1}$	$R_{2}$	*t*-Test
Ideal voltage regulator	8 kΩ	2 kΩ	1.74
Strong DLDO voltage regulator	8 kΩ	2 kΩ	1.11
Two DLDO sharing same VDD	500, 1 k, 2 k, 4 k, 8 kΩ	2 kΩ	0.36
Two-phase DLDO with 180 degree phase	500, 1 k, 2 k, 4 k, 8 kΩ	2 kΩ	0.43

**Table 2:** The comparison of this work with other methods where implementation is the method used and minimum number of traces for leakage is the number of traces where the *t*-test passes threshold 4.5.

	ASIC/FPGA	Implementation	Minimum Number of Traces for the Leakage (Higher Is Better)
[7]	ASIC	PDN	1k
[15]	ASIC	PDN	18k
[16]	ASIC	X	X
This work	ASIC	PDN	80k

**Table 3:** The *t*-test results of the power delivery grid network with different locations of the DLDO voltage regulator.

DLDO Voltage Regulator Position in Grid	max(|t|)	Correlation between Two Shares
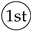	5.68	0.71
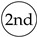	4.25	0.53
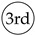	3.94	0.49
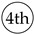	4.09	0.51
